# Cyclooxygenase-2 Glycosylation Is Affected by Peroxynitrite in Endothelial Cells: Impact on Enzyme Activity and Degradation

**DOI:** 10.3390/antiox10030496

**Published:** 2021-03-23

**Authors:** Sonia Eligini, Susanna Colli, Aida Habib, Giancarlo Aldini, Alessandra Altomare, Cristina Banfi

**Affiliations:** 1 Centro Cardiologico Monzino I.R.C.C.S., 20138 Milan, Italy; cristina.banfi@cardiologicomonzino.it; 2 Dipartimento di Scienze Farmacologiche, Università degli Studi di Milano, 20133 Milan, Italy; susanna.colli@unimi.it; 3 Department of Biochemistry and Molecular Genetics, Faculty of Medicine, American University of Beirut, Beirut 1107 2020, Lebanon; ah31@aub.edu.lb; 4 INSERM-UMR1149, Centre de Recherche sur l’Inflammation, and Sorbonne Paris Cité, Laboratoire d’Excellence Inflamex, Faculté de Médecine, Site Xavier Bichat, Université de Paris, 75018 Paris, France; 5 Dipartimento di Scienze Farmaceutiche, Università degli Studi di Milano, 20133 Milano, Italy; giancarlo.aldini@unimi.it (G.A.); alessandra.altomare@unimi.it (A.A.)

**Keywords:** cyclooxygenase-2, SIN-1, endothelial cell, N-linked glycosylation

## Abstract

The exposure of human endothelial cells to 3-morpholinosydnonimine (SIN-1) induced the expression of cyclooxygenase-2 (COX-2) in a dose- and time-dependent manner. Interestingly, after a prolonged incubation (>8 h) several proteoforms were visualized by Western blot, corresponding to different states of glycosylation of the protein. This effect was specific for SIN-1 that generates peroxynitrite and it was not detected with other nitric oxide-donors. Metabolic labeling experiments using ^35^S or cycloheximide suggested that the formation of hypoglycosylated COX-2 was dependent on de novo synthesis of the protein rather than the deglycosylation of the native protein. Moreover, SIN-1 reduced the activity of the hexokinase, the enzyme responsible for the first step of glycolysis. The hypoglycosylated COX-2 induced by SIN-1 showed a reduced capacity to generate prostaglandins and the activity was only partially recovered after immunoprecipitation. Finally, hypoglycosylated COX-2 showed a more rapid rate of degradation compared to COX-2 induced by IL-1α and an alteration in the localization with an accumulation mainly detected in the nuclear membrane. Our results have important implication to understand the effect of peroxynitrite on COX-2 expression and activity, and they may help to identify new pharmacological tools direct to increase COX-2 degradation or to inhibit its activity.

## 1. Introduction

Cyclooxygenase (COX), also known as prostaglandin (PG)H synthase, is the first enzyme in the conversion of fatty acid substrates, most notably arachidonic acid, to PGH_2_. PGH_2_ is further metabolized by downstream synthase enzymes to a range of prostanoids. Indeed, PGH_2_ is an unstable endoperoxide, the substrate for the terminal synthases as prostacyclin (PGI_2_)-synthase, PGE_2_-synthase or thromboxane A_2_ (TXA_2_)-synthase, which are responsible for the formation of the different prostanoids. COX is the major enzyme involved in their biosynthesis and two different isoforms, COX-1 and COX-2, with similar structure and catalytic activity, have been described [1]. Both isoforms are heme-containing glycoproteins mainly located on the inner and outer membranes of the nuclear envelope and on the lumenal surface of the endoplasmic reticulum [2]. The N-linked glycosylation, which occurs in the endoplasmic reticulum, is required for proper folding, activity, degradation, and turnover of the two proteins [3,4,5].

COX-1 and COX-2 show distinct expression and regulation. COX-1 is a ubiquitous protein, constitutively expressed in mammalian cells, having general housekeeping functions. Instead COX-2 is expressed at low level in resting cells but its synthesis is rapidly induced by stimuli such as free radicals, cytokines and growth factors, mostly in cells participating to the inflammatory response, such as monocytes, macrophages, and endothelial cells [6,7]. Therefore, it is mainly responsible for the production of prostanoids involved in pathological processes as acute and chronic inflammation and atherosclerosis [8,9,10].

Nevertheless, endothelial COX-2 also protects the cardiovascular system, although, in comparison with COX-1, it is only sparsely expressed in most areas of endothelium. Indeed, in mice with selective endothelial COX-2 knocked out, thrombosis is increased [11,12]. This effect is likely due to a reduction in PGI_2_ production, the major COX-2 metabolite synthesized by endothelial cells, which highlights a protective role in atherogenesis by reducing platelet activation, leukocyte adhesion, and smooth muscle cells proliferation [13,14].

One of the major causes of vascular inflammation and endothelial dysfunction is oxidative stress [15,16]. In particular, peroxynitrite produced in biological systems by the interaction of nitric oxide (NO) and superoxide anion, is a potent oxidant agent that can profoundly impair the endothelial functions. Despite its short half-life at physiological pH, the interaction of peroxynitrite with the cellular membrane and molecules with biological activity induces detrimental effects in pathophysiological oxidative stress conditions [17]. Indeed, following the exposure of biological systems to exogenous peroxynitrite, several cellular alterations occurs, including inhibition, inactivation or activation of enzymes, modification in protein structure, and disorders in cellular energetic balance and signaling pathways [18].

In this regard, it has been shown that peroxynitrite can provide the peroxide tone necessary for the activation of COX-1 and COX-2 resulting in an increased prostaglandin production [19]. However, peroxynitrite may also inhibit COX activity though tyrosin385 nitration, and thus divergent hypothesis regarding activation or inhibition of prostanoid synthesis are reported [20].

Herein, we investigated the effects of peroxynitrite generated by SIN-1 on COX-2 expression, activity, and degradation in human endothelial cells. For the first time, we evidenced that, peroxynitrite by interfering with an early step within the glycolytic pathway essential for protein N-linked glycosylation, induces post-translational modification of COX-2, resulting in a reduction of its activity and an acceleration of its degradation.

## 2. Materials and Methods

### 2.1. Cell Culture and Treatment

Primary cultures of human umbilical vein endothelial cells (HUVEC) were cultured in M199 (Lonza, Euroclone, Pero, Milan, Italy) supplemented with 10% heat-inactivated human AB serum, as described [21]. Cell treatment was carried out in M199 medium supplemented with 0.75% bovine serum albumin (BSA, fatty acid-free and low endotoxin, Merck, Milan, Italy) and 1% fetal calf serum (Lonza, Euroclone, Pero, Milan, Italy).

### 2.2. Cytotoxicity Assay

Cell viability was evaluated using neutral red reduction assay [22] and calculated as follows: relative viability = [(*Ae* − *Ab*)/(*Ac* − *Ab*)] × 100, where *Ab* is the background absorbance, *Ae* is the experimental absorbance, and *Ac* is the absorbance of controls. The various reagents did not affect cell viability at the concentration tested after 24 h incubation

### 2.3. Peroxynitrite Generation

The generation of peroxynitrite was evaluated using peroxynitrite assay kit (Abcam, Prodotti Gianni, Milan, Italy) according to the manufacturer’s instructions.

### 2.4. RNA Isolation and Analysis

Confluent HUVEC plated in 6-well plates were incubated 6 h in M199 medium supplemented with 0.75% bovine serum albumin and 1% fetal calf serum in the presence or in the absence of SIN-1 (Cayman Chemical, Cabru, Arcore, Milan, Italy). Total RNA was isolated with TRIzol reagent, and mRNA expression was assessed as previously described [23]. GAPDH mRNA was used as a control of mRNA loading. 

### 2.5. Western Blot Analysis

After the incubation of HUVEC with SIN-1, or IL-1α, or both the stimuli in combination, cells were harvested in lysis buffer pH 6.8, as described [23]. Cell debris was removed by centrifugation (10,000× *g* for 5 min) and protein content was measured with BCA protein assay. Proteins were separated by 7% SDS-PAGE gel and transferred to nitrocellulose membranes. The membranes were incubated overnight at 4 °C with primary antibodies against either COX-1 (1:500, Cayman Chemical, Cabru, Arcore, Milan, Italy), COX-2 (1:10,000), or β-actin (1:10,000, Merck). Blots were incubated with horseradish peroxidase-linked anti-mouse IgG antibody (1:5,000, Jackson ImmunoResearch Labs Inc., Li StarFISH, Cernuscosul Naviglio, Milan, Italy). Bands were visualized by enhanced chemiluminescence using the ECL kit (GE Healthcare, Milan, Italy) and quantified by means of a densitometer (GS800; Bio-Rad, Segrate, Milan, Italy) using the image analysis software QuantityOne (version 4.5.2; Bio-Rad, Segrate, Milan, Italy). The intensity of the bands relative to COX-2 after quantification, and background subtraction, was normalized to β-actin.

### 2.6. COX-2 Immunoprecipitation

HUVEC were lysed in lysis buffer (50 mM Tris-HCl pH 6.8, 30 mM n-octyl-β-D-glucopyranoside, 1 mM EDTA, 1 mM benzamidine). Antibody against COX-2 was added to protein A-Sepharose beads (Santa Cruz Biotechnology, Inc., D.B.A. Italia S.R.L., Segrate, Milan, Italy) and incubated with gentle rocking overnight at 4 °C. 0.2 mg of cellular lysate was added to the protein A-Sepharose beads for 2 h at 4 °C. After two washings, the pellet was lysed in Laemmli buffer and immunoprecipitated protein was separated on 7% SDS-PAGE gels and immunoblotted.

### 2.7. Endoglycosidase H and PNGase F Digestion

First, 20 μg of the immunoprecipitated COX-2 were treated with 25 μL of Na citrate buffer (50 mM, pH 6.2) containing 100 mM 2-mercaptoethanol and 0.1% SDS and heated for 10 min at 90 °C to denature the protein. After centrifugation at 10,000× *g*, the solubilized, denatured protein was treated with Na citrate buffer containing 25 mU/mL endoglycosidase H and 0.5 mM pefabloc. Samples were incubated overnight at 30 °C. For PNGase F digestion, 20 μg of immunoprecipitated proteins were denaturated and incubated with 25 mU/mL PNGase F (New England Biolabs, Euroclone, Pero, Milan, Italy) at 37 °C for 1 h as indicated in manufactured instructions. Digested protein was lysed in Laemmli buffer, analyzed on 7% SDS-PAGE gels and immunoblotted with antibody against COX-2.

### 2.8. Metabolic Labeling

Confluent cells were incubated in M199 medium and exposed either to SIN-1 or IL-1β alone or in combination. After 6 or 12 h incubation, 0.3 mCi of Tran [^35^S]-label (1077 Ci/mmol) (MP Biomedicals, DBA Italia, Milan, Italy) was added (0.1 mCi/mL) and cells were incubated for additional 6 or 12 h. After two washes, cells were lysed in ice-cold lysis buffer, and after centrifugation at 10,000× *g* for 10 min at 4 °C, COX-2 was immunoprecipitated as described above. Immunoprecipitated proteins were lysed in Laemmli buffer and separated on 7% SDS-PAGE gels. After exposure to Kodak films, bands were acquired by densitometer (GS800; Bio-Rad) using the image analysis software QuantityOne (version 4.5.2; Bio-Rad, Segrate, Milan, Italy).

### 2.9. Total Hexokinase Activity

After incubation of confluent HUVEC with the stimuli for 18 h, cells were lysed in buffer (20 mM K_2_HPO_4_, 1 mM EDTA, 5 mM DTT, 110 mM KCl, containing 10 mg/mL leupeptin, 1 mM benzamidine, 1 mM PMSF). Cellular lysates were centrifuged at 12,000× *g* for 10 min at 4 °C and the total hexokinase activity was measured by the conversion of NAD+ to NADH in a glucose-6-phosphate dehydrogenase-coupled reaction. Incubation medium for hexokinase activity measurements contained 50 mM Hepes/HCl (pH 7.6), 100 mM KCl, 7.4 mM MgCl_2_, 0.05% BSA, 5 mM ATP, 0.5 mM NAD+, 2.5 mM DTT, 0.5 mM D-glucose, 1 U/mL glucose-6-phosphate dehydrogenase (*Saccharomyces cerevisiae*). The reaction was performed at 37 °C for 90 min and stopped by the addition of 500 mM NaHCO_3_ (pH 9.4). NADH concentration was measured by fluorimeter at 340 nm excitation and 460-nm emission. Protein concentration was quantified by the Pierce BCA protein assay and hexokinase activity was expressed as U/mg protein/min.

### 2.10. Prostaglandins Measurement

Following treatment with SIN-1 or IL-1α either alone or in combination for 18 h, cells were washed twice and incubated with arachidonic acid (10 μM) or PGH_2_ (1 μM) for additional 30 min in Hank’s buffer (pH 7.4) containing 1 mg/mL BSA. In addition, prostaglandins production from endogenous substrate was measured after the exposure of HUVEC to the calcium ionophore A23187 (2 μM) for 30 min.

6-keto-PGF_1α_, PGE_2_, PGF_2α_, in cell culture supernatants were measured using enzyme immunoassay (EIA) kits (Cayman Chemical, Cabru, Arcore, Milan, Italy), according to the manufacturer’s instructions.

PGE_2_ production was also measured after the selective immunoprecipitation of COX-2. Immunoprecipitate was resuspended in 50 mM Tris HCl (pH 8.0) containing 1 mM phenol and 1 mg/mL BSA and preincubated at 37 °C with 1 mM hematin for 1 min as described previously [24]. The reaction was continued for 10 min after the addition of 25 μM arachidonic acid, and stopped by adding 1 volume of ice-cold enzyme immunoassay (EIA) buffer. The mixture was centrifuged for 1 min at 5000 g, and PGE_2_, the breakdown product of PGH_2_ in this reaction, was then measured in the supernatant by EIA.

### 2.11. Analysis of COX-2 Protein Degradation

COX-2 was induced in HUVEC following incubation with SIN-1 or IL-1α alone or in combination. After 18 h, the cells were treated with 2 μg/mL cycloheximide (CHX) to block the de novo synthesis of the protein, and HUVEC were harvested at different times (60, 120, 240, 360 min). The COX-2 protein was then detected by Western blot analysis.

### 2.12. Immunofluorescence Staining

After incubation of HUVEC with SIN-1 or IL-1α alone or in combination, cells were fixed in 2% paraformaldehyde, for 20 min at room temperature. Non-specific reactive sites were blocked with 5% bovine serum albumin solution containing 0.1% saponin (30 min, at room temperature). Cells were incubated overnight at 4 °C with 1 µg/mL antibody directed toward COX-2 and detection was performed with anti-mouse Alexa Fluor 488 (60 min, at room temperature) (Life Technologies Italia, Monza, Monza Brianza, Italy). The negative control was obtained omitting the primary antibody. Fluorescent images were acquired on Apotome microscope (Carl Zeiss S.p.A., Milan, Italy) at 40× of magnification using the image processor ZEN 2 (Carl Zeiss S.p.A., Milan, Italy).

### 2.13. Statistical Analysis

Results are expressed as mean ± SD. Statistical significance (*p* < 0.05) was evaluated by ANOVA with comparison between groups using the Tukey test with the GraphPad Prism software v 5.0.

## 3. Results

### 3.1. SIN-1 Induces COX-2 Expression in HUVEC

**Figure 1:**
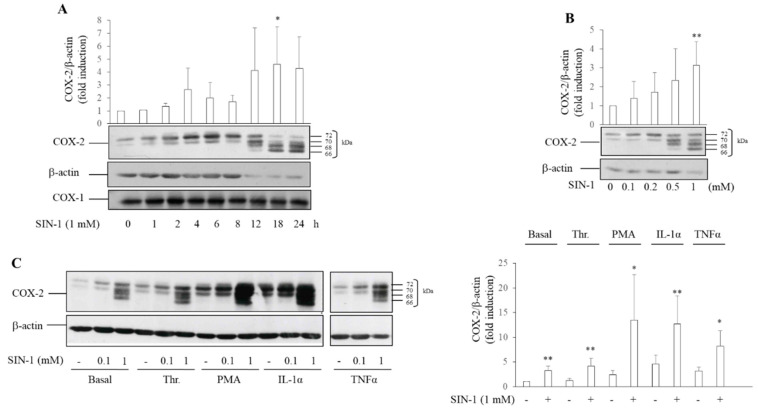
COX-2 expression induced by SIN-1. Human umbilical vein endothelial cells (HUVEC) were incubated with SIN-1 alone or in combination with stimuli such as thrombin (Thr), phorbol 12-myristate 13-acetate (PMA), TNFα, or IL-1α, and the protein expression was evaluated by Western blot analysis. β-actin was used as a control of protein loading. Densitometry is shown in the bar graph. (**A**) Time-dependent expression of COX-2 and COX-1 induced after exposure to 1 mM SIN-1. Results are representative of 3–6 independent experiments; * *p*< 0.05 vs. control cells treated with vehicle (**B**) Concentration-dependent expression of COX-2 induced by SIN-1 after 18 h incubation. Results are representative of 7 independent experiments; ** *p*< 0.01 vs. control cells (**C**) COX-2 protein levels in HUVEC exposed to 0.1 and 1 mM SIN-1 alone or in combination with 0.5 U/mL Thr, 5 nM PMA, 100 U/mL TNFα, or 25 U/mL IL-1α. The lanes for TNFα were run on a parallel gel. Results are representative of 5–9 independent experiments. * *p*< 0.05, ** *p*< 0.01 vs. respective cells incubated in the absence of SIN-1.

We have previously shown that SIN-1 dose-dependently induces in HUVEC, up to 6 h, an increase in the level of COX-2 protein, recognized as a double band (70–72 kDa), by a monoclonal antibody direct against the carboxyl-terminal region of the protein [23]. At first, the increase of COX-2 protein levels was attributed to mRNA accumulation (Supplementary Figure S1). Herein, we show that COX-2 protein increased as early as 1 h after the addition of SIN-1 with progressive increases of its levels upon 24 h incubation (Figure 1A). By contrast, the levels of COX-1, as well as its electrophoretic mobility, remained unaltered (Figure 1A). Of interest, when incubation was prolonged after 8 h, COX-2 was characterized by the presence of two additional bands at lower molecular weight (66 and 68 kDa, approximately) (Figure 1A). The appearance of multiple bands was dependent upon SIN-1 concentration (Figure 1B), reaching the maximum at 1 mM, and was not the result of a decrease in cell viability as tested by neutral red assay (data not shown).

The presence of multiple bands was also observed when HUVEC were exposed for 18 h to 1 mM SIN-1 in combination with agents known to induce COX-2 in HUVEC, such as thrombin, TNFα, phorbol 12-myristate 13-acetate (PMA), and IL-1β (Figure 1C).

**Figure 2:**
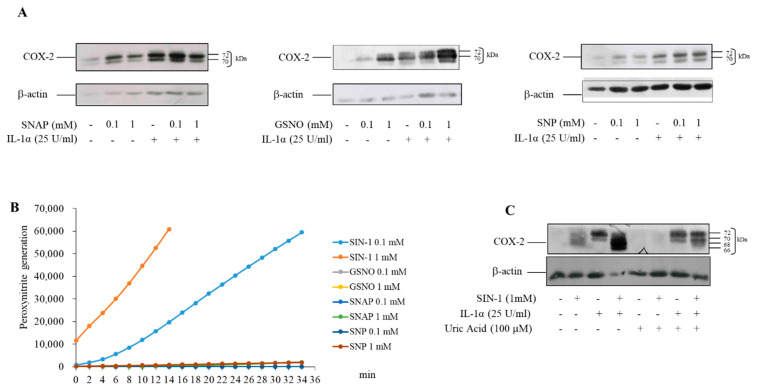
COX-2 expression induced by NO-donors. (**A**) HUVEC were incubated for 18 h with different concentrations of SNAP, GSNO, SNP alone or in combination with IL-1α. (**B**) The generation of peroxynitrite was monitored by the fluorescence measured at Ex/Em = 490/530 nm using the peroxynitrite assay kit (**C**) HUVEC were preincubated with 100 μM uric acid for 1 h. Incubation was continued for 18 h after the addition of 1 mM SIN-1. COX-2 expression was evaluated by Western blot analysis. β-actin was used as a control of protein loading. Results are representative of 3 independent experiments.

Other NO donors such as S-nitroso-N-acetyl-DL-penicillamine (SNAP), S-nitrosoglutathione (GSNO), sodium nitroprusside (SNP), alone or associated with IL-1α, induced COX-2 in HUVEC, but in this case the protein did not show the additional bands observed with SIN-1 (Figure 2A). Unlike these NO-donors, SIN-1 spontaneously releases NO and superoxide anion, which react to form peroxynitrite when added to HUVEC (Figure 2B). Uric acid, a peroxynitrite scavenger, completely prevented COX-2 induction by SIN-1, indicating that this short-lived oxidant anion, generated by the decomposition of SIN-1, was responsible for the COX-2 induction (Figure 2C). Further, molsidomine, the inactive drug precursor of SIN-1, did not induce the formation of multiple proteoforms of COX-2 (Supplementary Figure S2).

### 3.2. COX-2 Induced by SIN-1 Is Hypoglycosylated

**Figure 3:**
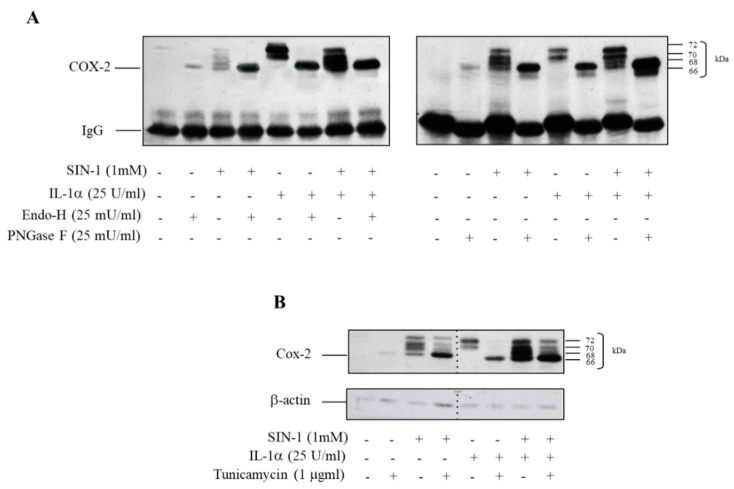
SIN-1 alters the glycosylation pattern of COX-2. HUVEC were incubated for 18 h with SIN-1 alone or in combination with IL-1α. (**A**) After immunoprecipitation, endoglycosidase H, which cleaves high-mannose-type and hybrid oligosaccharide chains, or PNGase F, which cleaves N-linked tri- and tetraantennary complex-type chains, were added. (**B**) Incubation of cells is performed in the presence of tunicamycin. COX-2 expression was evaluated by Western blot analysis. β-actin was used as a control of protein loading. The lanes were run on the same gels but were non-contiguous. Results are representative of 3 independent experiments.

As already mentioned, COX-2 is characterized by the presence of multiple glycosylation sites [3]. To assess whether SIN-1 had an impact on the glycosylation pattern of COX-2, the protein was immunoprecipitated from cell lysates harvested upon incubation with SIN-1 alone or in combination with IL-1α for 18 h and incubated either with endoglycosidase H, which cleaves high-mannose-type and hybrid oligosaccharide chains [25] or with PNGase F, which cleaves N-linked tri- and tetraantennary complex-type chains [26]. Both these treatments completely prevented the appearance of the pattern induced by SIN-1, resulting in a single band characterized by the lowest molecular weight (Figure 3A). These results confirm that the different proteoforms were due to differences in glycosylation. Moreover, experiments performed by using tunicamycin, which prevents COX-2 N-glycosylation [3], showed that the glycosylation of COX-2, induced either by SIN-1 or by IL-1α, was prevented and resulted in a major single band of approximately 66 kDa (Figure 3B), which represents the apparent molecular weight of unglycosylated COX-2. All together, these results suggest that COX-2 induced by SIN-1 is characterized by an altered state of N-glycosylation.

**Figure 4:**
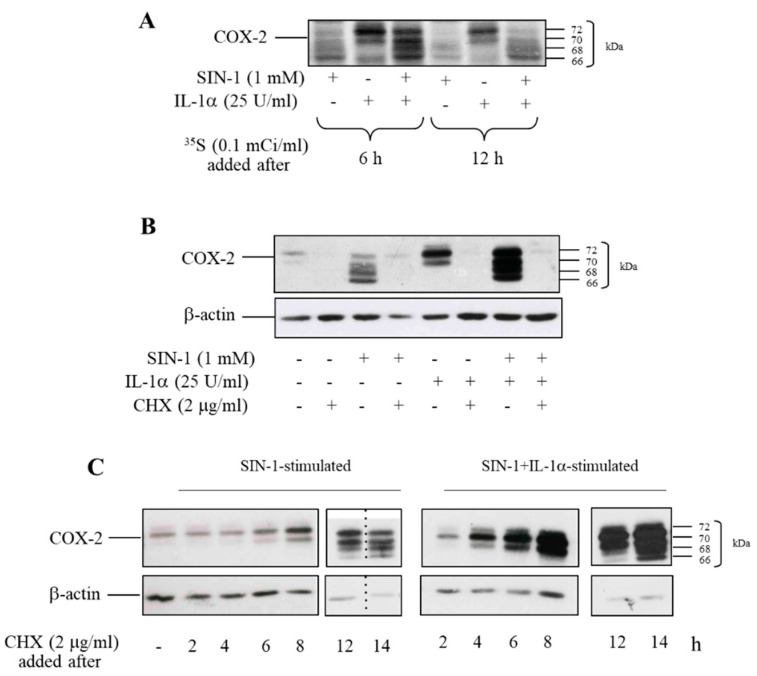
SIN-1 induces a de novo synthesis of hypoglycosylated COX-2. HUVEC were treated with SIN-1 or IL-1α, either alone or in combination for 18 h. (**A**) Tran [^35^S]-label was added 6 or 12 h after the beginning of incubation. At the end of incubation, COX-2 was immunoprecipitated. (**B**) Cells were treated with the stimuli for 18 h in the presence of cycloheximide (CHX). (**C**) CHX was added to HUVEC incubated with SIN-1 or SIN-1 + IL-1α, either alone or in combination, at different time after the start of incubation, as indicated. COX-2 expression was evaluated by Western blot analysis. β-actin was used as a control of protein loading. Samples with CHX added for 12 and 14 h-treatment were run on separate parallel gels. For SIN-1-treated cells, the samples for the cells treated with CHX added for 12 and 14-h were run on the same gel but were non-contiguous. Results are representative of 3 independent experiments.

To assess whether the defective glycosylated COX-2 was synthesized as such or it was the consequence of the removal of sugar chains, protein labeling with [^35^S]-methionine was performed. Tran [^35^S]-label was added either 6 or 12 h after the beginning of incubation with SIN-1 alone or in combination with IL-1α. Incubation lasted 18 h and COX-2 was immunoprecipitated thereafter. When Tran [^35^S]-label was added after 6 h, the radioactive profile was superimposable to variation in immunoreactivity observed by Western analysis. By contrast, when the radioactive reagent was added after 12 h, more labelling of the lower bands was observed (Figure 4A). This data indicates that nascent COX-2 induced by a prolonged exposure of HUVEC to SIN-1 is likely to lack glycosylation.

This finding was confirmed by experiments carried out with cycloheximide (CHX) added at different time points after cells exposure to SIN-1 alone or in combination with IL-1α. The simultaneous addition of CHX with the stimuli completely prevented COX-2 induction (Figure 4B). When CHX was added at early incubation times (i.e., less than 8–12 h), a time frame that allows the synthesis of the protein correctly glycosylated, the appearance of additional bands was not detected (Figure 4C). Beyond this period, the newly synthesized COX-2 showed an altered glycosylation pattern, indicating that deglycosylation of the protein is unlikely to occur, and supported the notion that the formation of additional bands depends upon the delayed synthesis of a defective glycosylated COX-2.

### 3.3. The Effect of SIN-1 on COX-2 Involves Hexokinase Activity

**Figure 5:**
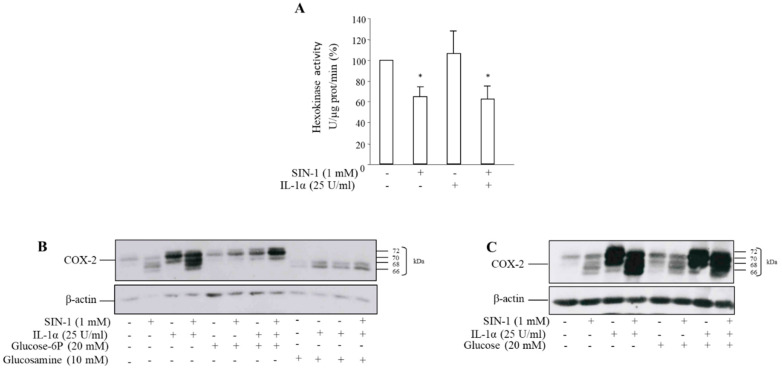
SIN-1 reduces the hexokinase activity. HUVEC were treated with SIN-1 or IL-1α either alone, or in combination for 18 h. (**A**) Hexokinase activity was measured by fluorimetry through the conversion of NAD^+^ to NADH in a glucose-6-phosphate dehydrogenase-coupled reaction. *n* = 4; * *p* < 0.05 vs. cells incubated in the absence of SIN-1. Cells were treated with the stimuli in the presence of (**B**) glucose-6P or of the hexokinase inhibitor glucosamine, or (**C**) glucose. COX-2 expression was evaluated by Western blot analysis. β-actin was used as a control of protein loading. Results are representative of 3 independent experiments.

N-glycosylation of proteins occurs through the formation of a lipid-glycan precursor that is bound to dolichol phosphate [27]. Glucose is the central monosaccharide in carbohydrate metabolism, and it can be converted to glucose-6-P by hexokinase leading to the formation of all other sugars relevant in the glycosylation process [28]. To investigate the involvement of this enzyme in the expression of aglycosylated COX-2, the hexokinase activity was measured. After exposure of HUVEC to 1 mM SIN-1 for 18 h, total cellular hexokinase activity was significantly reduced with respect to control, as well as after incubation with IL-1α plus SIN-1 (Figure 5A). The synthesis of a hypoglycosylated COX-2 induced by SIN-1 can be prevented by the addition to the medium of glucose-6-phosphate (Figure 5B). In addition, COX-2 induced by IL-1α in the presence of glucosamine, a competitive inhibitor of hexokinase that prevents the addition of N-linked oligosaccharides to COX-2, produced a similar shift toward a lower molecular mass of COX-2 similar to what observed with SIN-1 (Figure 5B). By contrast, no effect was observed in the presence of glucose (Figure 5C).

Since dolichol phosphate, as above cited, is essential for protein N-glycosylation [27], we assessed the involvement of dolichol biosynthetic pathway in the synthesis of COX-2 induced by SIN-1. HUVEC were incubated in the presence of 100 μM mevalonate that is the precursor of dolichol formation. In these experimental conditions, COX-2 induced by SIN-1 was hypoglycosylated, thus excluding an effect of SIN-1 on mevalonate/dolichol pathway (data not shown).

### 3.4. SIN-1 Impairs Prostaglandins Production in HUVEC

**Figure 6:**
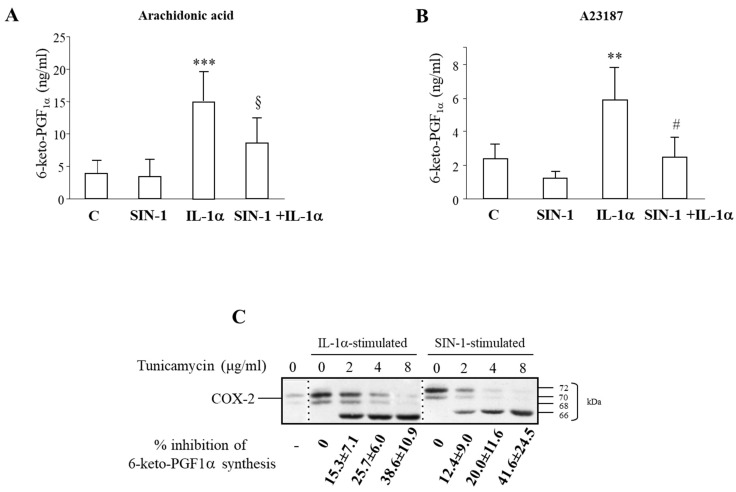
SIN-1 inhibits the prostaglandin production. HUVEC were treated for 18 h with SIN-1 or IL-1α either alone or in combination. (**A**) 6-keto-PGF_1α_ production was measured after the exposure of cells to 10 μM arachidonic acid for 30 min. *n* = 17 independent experiments; *** *p* < 0.001 vs. unstimulated cells; § *p* < 0.001vs. IL-1α stimulated cells). (**B**) Prostaglandin production was measured after the exposure of cells to 2 μM calcium ionophore A23187 for 30 min; *n* = 5; ** *p* < 0.01 vs. unstimulated cells; # *p* < 0.01vs. IL-1α. (**C**) HUVEC were incubated with SIN-1 or IL-1α in the presence or absence of different concentration of tunicamycin for 6 h and Western blot analysis was performed. The lanes were run on the same gel but were non-contiguous. Results are representative of 4 independent experiments. 6-keto-PGF_1α_ levels were measured by EIA.

The increased COX-2 levels detected after HUVEC incubation with SIN-1 for 18 h, were not associated with augmented levels of 6 keto-PGF_1α_, measured in the medium of cells after the addition of arachidonic acid. By contrast, a marked increase of 6-keto-PGF_1α_ levels were measured in medium of IL-1α stimulated cells. Moreover, the co-incubation of SIN-1 with IL-1α decreased 6-keto-PGF_1α_ levels with respect to those elicited by IL-1α alone (Figure 6A), thus suggesting that SIN-1induced a significant increase in the accumulation of a defective COX-2.

In order to evaluate the impact of SIN-1 on the conversion of arachidonic acid from an endogenous source, cells were also incubated with the Calcium ionophore A23187. Although 6-keto-PGF_1α_ levels were lower than those measured in the presence of the exogenously added substrate arachidonic acid, results showed the same trend (Figure 6B). Measurement of the levels of PGE_2_ and PGF_2α_, revealed that also these PGs were decreased in the presence of SIN-1, performed in both experimental conditions (data not shown).

The effect of SIN-1 on the activity of PGI_2_-synthase, which is upstream of 6-keto-PGF1α, was then addressed. We have previously shown that PGI_2_-synthase activity was not affected in HUVEC incubated with SIN-1 for 6 h [23]. By contrast, a prolonged incubation (18 h) with SIN-1 significantly reduced the basal capacity of cells to convert PGH_2_ to 6-keto-PGF_1α_. In these conditions, IL-1α did not modify prostacyclin synthesis as previously described [24] (76.0 ± 20.6 and 47.0 ± 7.4 ng/mL 6-keto-PGF_1α_ for unstimulated cells and after incubation with SIN-1 respectively *n* = 5 *p* < 0.05; 74.40 ± 15.4 and 46.2 ± 10.9 ng/mL 6-keto-PGF_1α_ for IL-1α and IL-1α + SIN-1 respectively, *n* = 5 *p* < 0.05).

The contribution of glycosylation on the COX-2 activity was confirmed using tunicamycin that has been previously reported to inhibit prostaglandin production in several cell types [29,30]. Tunicamycin concentration-dependently reduced 6-keto-PGF_1α_ production in HUVEC treated with IL-1α for 6 h. The same effect was detected also when COX-2 was induced by SIN-1. In these conditions, COX activity, evaluated in term of the production of 6-keto-PGF_1α_, was further reduced by tunicamycin. The impairment of enzymatic activity went in parallel with the appearance of the 66 kDa aglycosylated COX-2 (Figure 6C). The lack of hypoglycosylated bands in COX-2 protein after SIN-1 stimulation is ascribed to the short period time of incubation (6 h instead of 18 h). Longer incubation time is indeed not possible due to the toxicity of tunicamycin in our experimental model.

### 3.5. Partial Recovery of COX-2 Activity Impaired by SIN-1

**Figure 7:**
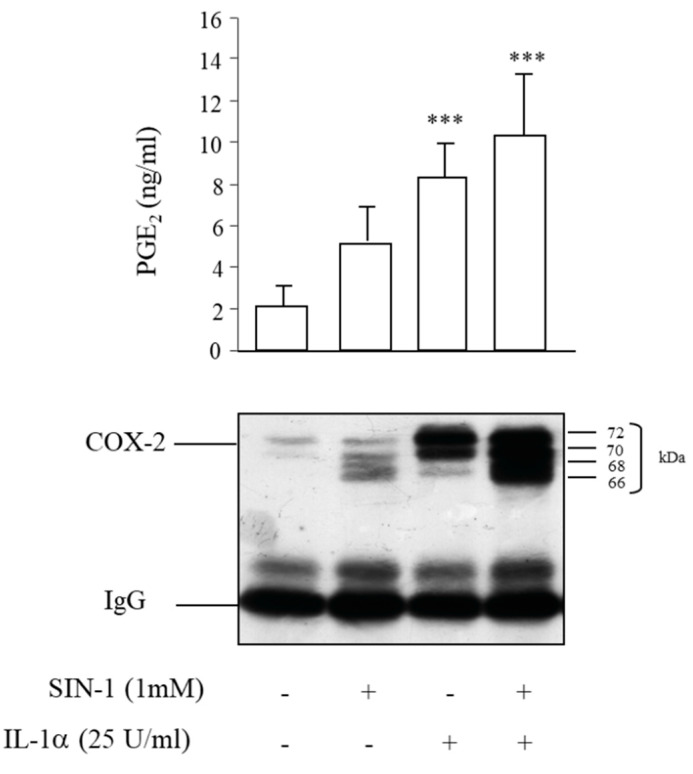
COX-2 activity is partially recovered after immunoprecipitation of the enzyme. HUVEC were treated with SIN-1 or IL-1α either alone or in combination for 18 h and then COX-2 was immunoprecipitated. PGE_2_ production was measured after the exposure of immunoprecipitates to 25 μM arachidonic acid for 10 min and determined by EIA. *n* = 6 independent experiments; *** *p* < 0.001 vs. unstimulated cells.

After cell stimulation, COX-2 was immunoprecipitated and incubated with arachidonic acid in the presence of hematin, which replaces the heme group. In this condition the levels of PGE_2_, which reflect COX-2 activity, being the product of the PGH_2_ hydrolysis, were slightly increased in the immunoprecipitates of cells treated with SIN-1 compared to the corresponding controls. Therefore, after the replacement of the prosthetic group, the COX-2 activity was partially recovered, suggesting an additional effect of SIN-1 due, at least in part, to the interaction with the heme group (Figure 7).

### 3.6. SIN-1 Accelerates the Turnover of Hypoglycosylated COX-2

**Figure 8:**
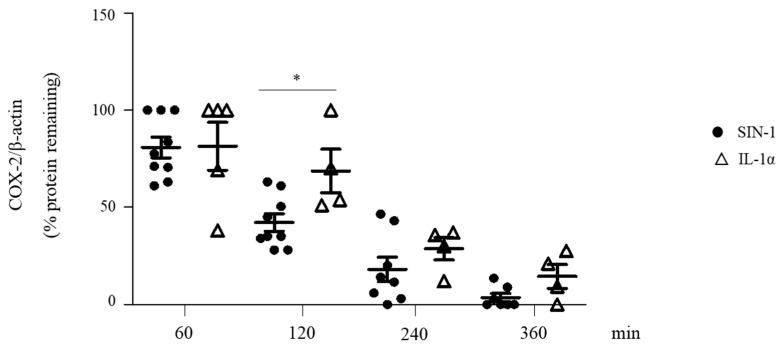
SIN-1 accelerates COX-2 degradation. HUVEC were treated with SIN-1 or IL-1α for 18 h. After the addition of 2 μg/mL cycloheximide (CHX), cells were incubated for different times (60, 120, 240, 360 min) and then harvest in lysis buffer. COX-2 expression was evaluated by Western blot analysis. β-actin was used as a control of protein loading. *n* = 4–9 independent experiments; * *p* < 0.05.

It is known that the status of COX-2 glycosylation affects the turnover of the protein [4,31]. Experiments of protein decay performed in SIN-1- or IL-1α-stimulated HUVEC showed that COX-2 induced by SIN-1 had a more rapid decay compared to that of protein induced by IL-1α, suggesting that glycosylation affects the COX-2 turnover (Figure 8).

### 3.7. Altered Intracellular Localization of Hypoglycosylated COX-2

**Figure 9:**
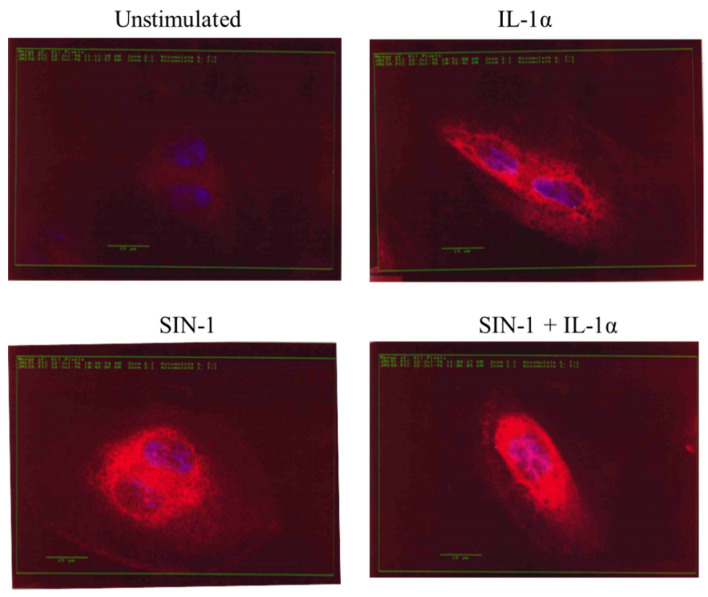
SIN-1 affects the intracellular COX-2 localization. HUVEC unstimulated or treated with IL-1α or SIN-1 alone or in combination for 18 h, were fixed in 2% paraformaldehyde and stained for COX-2 (in red). Nuclei (in blue) were visualized by Hoechst 33258. Images (400 x magnification) are representative of 3 independent experiments.

COX-2 is poorly expressed in quiescent HUVEC so it was not visualized in unstimulated cells (Figure 9). After 18 h incubation of HUVEC with IL-1α a marked increase of COX-2 immunoreactivity was detected along the nuclear membrane and especially in the cytoplasmic compartment. In contrast, the stimulation of HUVEC with SIN-1 induced a protein localization mainly in the nuclear membrane. This peculiar intracellular distribution was also visualized after the incubation of cells with SIN-1 plus IL-1α (Figure 9).

## 4. Discussion

In this paper, we provide evidence that SIN-1, by generating peroxynitrite, induces the expression of an inactive hypoglycosylated proteoform of COX-2. Indeed, we showed that prolonged (>8 h) treatment of HUVEC with SIN-1, induced a de novo synthesis of COX-2 mainly localized in the nuclear membrane, and characterized by hypoglycosylation, and loss of activity.

Of interest, molsidomine, the precursor of SIN-1, has been used as a long-acting antianginal drug for more than 30 years. In addition to its vasodilating effect, it has been shown that SIN-1 is also able to inhibit platelet aggregation [32]. However, in vitro and in vivo studies have excluded an effect of SIN-1 on COX metabolic pathway [33]. This observation is in accordance with the induction of an inactive enzyme as we have found. Vascular endothelium plays a pivotal role in maintaining cardiovascular homeostasis through the synthesis and release of several vasoactive mediators. An imbalance between vasorelaxant and vasoconstrictor factors leads to endothelial dysfunction, which is at the origin of cardiovascular diseases, such as atherosclerosis and its thrombotic complications [34]. The role of COX-2 in atherothrombosis is very complex and not fully understood. Tang et al. have indeed demonstrated that the depletion of endothelial COX-2 in LDLr knockout mice inhibits the synthesis of PGI_2_, PGE_2_, elevates blood pressure, and enhances atherogenesis [12]. Further, it has been shown that in physiological conditions COX-2 protects blood vessels against atherosclerotic lesions, independently of local production of PGI_2_ [35]. Additionally, stimuli involved in the onset and progression of atherosclerosis, like cytokines, free radicals, growth factors, and hypoxia, induce COX-2 expression, which via PGI_2_ production, contributes to preserving the vessel wall from injury [36].

Although it is known that peroxynitrite induces COX-2 expression in vitro and in vivo [23,37], we show, in this study, that prolonged exposure of HUVEC to SIN-1 provokes the synthesis of an abnormal COX-2, characterized by an incomplete glycosylation. Indeed, SIN-1 induced an enzyme with different states of glycosylation, at 72, 70, 68, and 66 kDa. Several studies have showed the induction of inflammatory mediators in different types of cells after exposure to SIN-1, but no information however is available about its effect on the glycosylation status of the proteins induced [38,39,40]. The induction of a hypoglycosylated form of COX-2 was dependent on peroxynitrite generated by SIN-1, because, uric acid, a peroxynitrite scavenger, completely prevented the induction of hypoglycosylated COX-2. Of note, in our experimental system, the SIN-1-derived peroxynitrite selectively affected only COX-2, but not COX-1. Further, the expression of hypoglycosylated COX-2 was induced by SIN-1 only, because this effect was not obtained with other NO-donors, such as SNAP, GSNO, and SNP, which indeed induced a properly glycosylated COX-2.

The glycosylation process is crucial for the synthesis, folding, stability, localization and activity of many proteins [27]. COX-2 enzyme has five potential sites for N-glycosylation within its structure (Asn53, Asn130, Asn396, Asn580, and Asn592), three of which are usually glycosylated [3,41]. The fifth site is rarely glycosylated, and the fourth site is partially glycosylated, thus leading to proteoforms of 72 and 74 KDa [4]. While the glycosylation sites at Asn53, Asn130, and Asn396 are required for a proper folding of the enzyme, the glycosylation at Asn580 affect the COX-2 activity by controlling the enzyme’s turnover [4]. In this paper we have not investigated which aminoacidic residue is affected by SIN-1 treatment, and, therefore our finding deserves further investigations. However, the appearance of four bands after exposure to SIN-1 suggests that all the potential sites for N-glycosylation are affected.

Our finding that SIN-1 induced a hypoglycosylated COX-2 enzyme was verified by means of experiments performed with tunicamycin that prevents N-linked glycosylation and with endoglycosidase H and PNGase F, which remove the high-mannose-type N-linked and tri- and tetraantennary complex-type chains, respectively. In these experimental conditions, only an aglycosylated COX-2 proteoform, having a molecular weight of 66 KDa was detectable.

The process of COX-2 glycosylation implicates the attachment of the oligosaccharide to the protein, through N-linkage on asparagine [41]. The first step of this process involves the assembly of the glycan precursor, composed of monosaccharide units attached to dolichol, the oligosaccharide substrate for the N-linked protein glycosylation in the endoplasmic reticulum [27]. The glucose metabolism supplies the substrate for the glycosylation, and for this process free glucose is phosphorylated by hexokinase enzyme to glucose 6-phosphate [42]. Our results show a significant reduction of the hexokinase activity after incubation of HUVEC with SIN-1. Moreover, the administration of glucose 6-phosphate led to the formation of COX-2 properly glycosylated, thus supporting the hypothesis that the inhibition of this step was responsible, at least in part, for a hypoglycosylated enzyme synthesis. This hypothesis was reinforced by the fact that glucosamine, a competitive inhibitor of hexokinase, produced a hypoglycosylated COX-2 quite similar to that observed with SIN-1. The mechanism by which SIN-1 affects the hexokinase activity is not investigated in this paper, however we can speculate a peroxynitrite-dependent tyrosine nitration as observed in chronic consumption of ethanol [43]. Moreover, we excluded that the dolichol biosynthetic pathway affected by SIN-1 treatment.

The effect of glycosylation on COX-2 activity is not fully understood. Indeed, it has been reported that the inhibition of glycosylation is associated with both a decrease and an increase of PGE_2_ production [31,44]. Experiments performed in the presence of tunicamycin, showed a progressive decrease of the activity that goes in parallel with the reduction of glycosylated status, suggesting that the glycosylation status affects the activity of the protein. Accordingly to our result it has been evidenced that the glycosylation of COX-2 is necessary to obtain an active protein [3].

Indeed, in our study, the hypoglycosylation of COX-2 induced by SIN-1 is associated with an impairment in the production of 6-keto-PGF_1α_, the stable breakdown product of PGI_2_. This result could be ascribed to an effect of SIN-1 on PGI_2_ synthase. Indeed, it has been reported a selective inhibition of PGI_2_ synthase by peroxynitrite dependent by nitration of Tyr-residue at the active site [45]. In a previous report, we did not detect a reduction of the prostaglandin production in HUVEC after 6 h incubation with SIN-1, and the conversion of exogenous PGH_2_ into 6-keto-PGF_1α_ was unchanged [23]. In contrast, herein, we show that prolonged exposure of HUVEC to SIN-1 affected the activity of PGI_2_ synthase, as detected by the reduced levels of 6-keto-PGF_1α_ released after exposure of the cells to PGH_2_. To increase the complexity of SIN-1 effect on HUVEC, the inhibition of the production of other PGs, such as PGE_2_ and PGF_2α,_ suggests that, in addition to PGI_2_, also COX-2 activity is impaired.

It has also been shown that peroxynitrite can differently modulate both COX-1 and COX-2 activity depending on its concentration: at low concentrations, it induces COX activities, whereas, at high concentrations, it inhibits the activity of both enzymes [46]. The mechanism through which peroxynitrite modulates COX activity is not clear, but an interaction with the Fe^2+^ in the heme group at the active site of the enzyme has been proposed [47]. In support to this hypothesis, we show that the enzyme activity, detected in the presence of hematin, to restore the prosthetic heme group, was partially recovered, thus suggesting that peroxynitrite also affects the prosthetic heme group of COX-2

Glycosylation is a post-translational modification that can also affect the turnover of COX-2. It has been demonstrated that the prevention of COX-2 glycosylation by glucosamine-hydrochloride increase the protein turnover [31]. Similarly, here we demonstrate that the hypoglycosylated COX-2 induced by SIN-1 is more rapidly degraded with respect to the protein induced by IL-1α. The mechanism involved has not been investigated in this paper, however, we can hypothesize that the altered proteins, as well as inactive proteins, are more sensitive to degradation than the native form. Indeed, structurally damaged proteins can be degraded via the endoplasmic reticulum-associated degradation system, which transports proteins from the endoplasmic reticulum to the cytoplasm for proteolysis by the 26S proteasome [48]. COX-2 degradation may be also mediated by a substrate-dependent suicide inactivation. This pathway requires a functional COX active site and begins from the suicide inactivated form of the protein [49]. Therefore, we cannot exclude that also this pathway is involved in the accelerated degradation of COX-2 induced by SIN-1. Indeed, it has been speculated that the substrate-dependent degradation may be a system to degrade COX-2 that accumulates into the endoplasmic reticulum and it is not required for the synthesis of prostaglandins in the reticulum [50].

Moreover, it has been shown that the post-translational glycosylation of Asn594 is the signal for the exit of glycosylated COX-2 from the endoplasmic reticulum, and a mutated form of COX-2 in which N-glycosylation is prevented, is retained in the reticulum. Although most of the COX-2 protein has been detected in the perinuclear envelope and in the endoplasmic reticulum, minimal amounts are localized in the Golgi apparatus and the translocation of COX-2 to the Golgi may represent an important step for the biosynthesis of PGE_2_. Indeed, it seems that cytosolic phospholipase A2, which provides the arachidonic acid for prostaglandin synthesis, preferentially translocates to the Golgi in response to calcium mobilization [50]. In contrast, COX-2 located in the perinuclear membrane seems to be mainly involved in the generation of PGI_2_ [51]. In our model, we detected a marked increase of immunostaining in the perinuclear and cytosolic compartments, after stimulation of HUVEC with SIN-1. However, if compared to COX-2 induced by IL-1α, COX-2 induced by SIN-1 is mainly located in the perinuclear compartment. This different subcellular distribution might be related to the different status in the cell cycle: in proliferating MSCP5 keratinocytes COX-2 has been shown to be located in the nuclear membrane and in the endoplasmic reticulum adjacent to the nucleus, whereas in resting MSCP5 keratinocytes a predominant localization in the nucleus was detected [52].

Whether transcription factors are involved in the effects induced by SIN-1 on COX-2, this has not been investigated in this study. It is known that COX-2 may be regulated by the activation of the oxidative-stress-responsive transcription factor NF-κB, known to mediate the expression of several genes involved in the inflammatory response [53]. However, despite it has been evidenced that peroxynitrite active NF-κB, an increase of COX-2 expression after exposure of endothelial cells to SIN-1 was not observed [54]. Thus, it has been hypothesized that other nuclear transcription factors might be involved in COX-2 expression, such as high-mobility group protein I (Y) [55].

Further, we have not assessed the effect of SIN-1 on the antioxidant levels in the cells. Peroxynitrite is indeed described as a potent oxidant agent with cytotoxic properties [56], which can oxidize and modify biological molecules such as lipids, proteins, and DNA, as well as depleting antioxidant defenses [57]. These antioxidant defenses include enzymes such as superoxide dismutase, catalase, glutathione peroxidase, glutathione reductase, and glucose-6-phosphate dehydrogenase, whose activities were significantly reduced after in vitro treatment with SIN-1 [58]. In addition, it has been observed that SIN-1 affects the intracellular redox balance, likely through a depletion of glutathione [59]. However, depending on the amount of peroxynitrite produced, the cell type, and the microenvironment, SIN-1 may induce a protective antioxidant response to oxidative stress. Indeed, it has been shown that SIN-1 activate the Nrf2 pathway, resulting in increased expression of target genes encoding for cytoprotective enzymes as NAD(P)H quinone oxidoreductase I, and for antioxidant enzymes as heme-oxygenase 1 [60].

## 5. Conclusions

In this paper, we show a novel mechanism by which peroxynitrite generated by SIN-1 decomposition affects the COX-2 N-linked glycosylation, leading to the expression of a hypoglycosylated protein characterized by reduced activity and increased turnover.

COX-2 and its prostanoids are involved in several human diseases as various human cancer [61,62] and numerous studies directed to highlight the mechanisms of regulation of its activity, have led to the development of several anti-inflammatory drugs and selective inhibitors that act through a direct inhibition of the enzyme. However, a reduction of COX-2 with agents that mediate its proteasomal degradation may represent an alternative mechanism to eliminate COX-2 protein in several disease. Moreover, more recently, the possibility to modulate its activity by post-translational modification such as glycosylation, phosphorylation, and N-nitrosylation has been hypothesized [63]. These new mechanisms governing COX-2 activity can pave the way to discover new therapeutic strategies. In this context, our finding provide new information about a posttranscriptional regulation of COX-2. Although further studies are required to better understand the mechanisms involved in COX-2 glycosylation and in the regulation of its activity by post-translational modulators, our contribution may help to provide the bases to identify new pharmacological tools that increase COX-2 degradation or inhibit its activity.

## Data Availability

Data collected in the study will be made available using the data repository Zenodo (https://zenodo.org accessed on 15 March 2021) with restricted access upon request to direzione.scientifica@ccfm.it.

## Supplementary Materials

The following are available online at https://www.mdpi.com/2076-3921/10/3/496/s1, Figure S1: COX-2 mRNA levels. Figure S2: Effect of Molsidomine on COX-2 expression.
