# Supplementary material for: Cyclooxygenase-2 Glycosylation Is Affected by Peroxynitrite in Endothelial Cells: Impact on Enzyme Activity and Degradation
Source: Antioxidants (Basel). 2021 Mar 23;10(3):496. doi: 10.3390/antiox10030496 (PMC8005028; doi:10.3390/antiox10030496)
Supplement: Supplementary file 1 [file antioxidants-10-00496-s001.pdf]

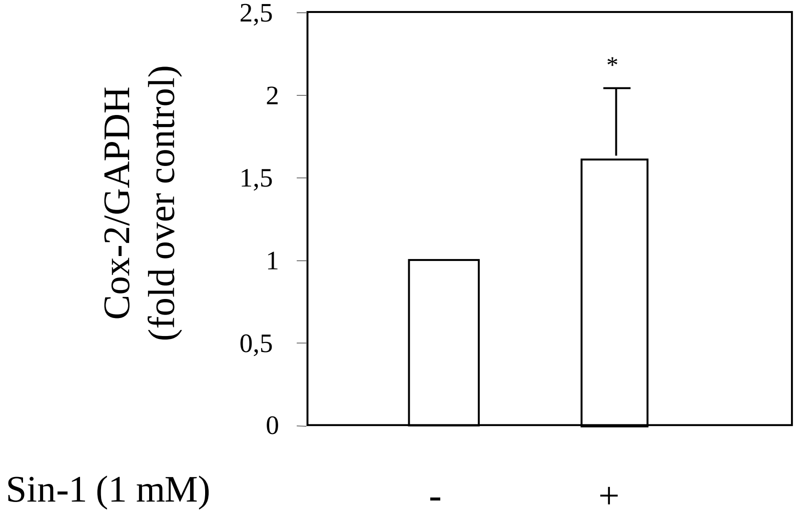

Figure S1. COX-2 mRNA levels. Cells were incubated with 1 mM SIN-1 for 6 h. COX-2 mRNA levels were evaluated by Northern analysis. GAPDH mRNA was used as a control of the mRNA loading. Results are representative of 6 independent experiments; *$p<0.05$.

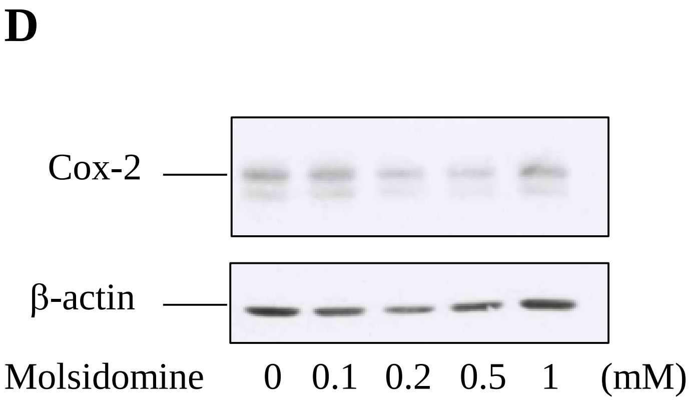

Figure S2. Effect of Molsidomine on Cox-2 expression. HUVEC were incubated for 18 hours with Molsidomine, the inactive precursor of SIN-1. Cox-2expression was evaluated by Western blot analysis. β-actin was used as a control of protein loading.

Supplemental Fig. 2
